# Male mice housed in groups engage in frequent fighting and show a lower response to additional bone loading than females or individually housed males that do not fight

**DOI:** 10.1016/j.bone.2013.01.029

**Published:** 2013-05

**Authors:** Lee B. Meakin, Toshihiro Sugiyama, Gabriel L. Galea, William J. Browne, Lance E. Lanyon, Joanna S. Price

**Affiliations:** a School of Veterinary Sciences, University of Bristol, Bristol BS40 5DU, UK; b Centre for Multilevel Modelling, University of Bristol, Bristol, UK

**Keywords:** Bone adaptation, Artificial loading, Mechanical strain, Physical activity, Housing

## Abstract

Experiments to investigate bone's physiological adaptation to mechanical loading frequently employ models that apply dynamic loads to bones *in vivo* and assess the changes in mass and architecture that result. It is axiomatic that bones will only show an adaptive response if the applied artificial loading environment differs in a significant way from that to which the bones have been habituated by normal functional loading. It is generally assumed that this normal loading is similar between experimental groups. In the study reported here we found that this was not always the case. Male and female 17-week-old C57BL/6 mice were housed in groups of six, and a single episode (40 cycles) of non-invasive axial loading, engendering 2,200με on the medial surface of the proximal tibiae in sample mice, was applied to right tibiae on alternate days for two weeks. This engendered an adaptive increase in bone mass in females, but not males. Observation revealed the main difference in behaviour between males and females was that males were involved in fights 1.3 times per hour, whereas the females never fought. We therefore housed all mice individually. In females, there was a similar significant osteogenic response to loading in cortical and trabecular bone of both grouped and individual mice. In contrast, in males, adaptive increases in the loaded compared with non-loaded control bones was only apparent in animals housed individually. Our interpretation of these findings is that the frequent vigorous fighting that occurs between young adult males housed in groups could be sufficient to engender peak strains and strain rates that equal or exceed the stimulus derived from artificial loading. This indicates the importance of ensuring that physical activity is consistent between groups. Reducing the background level of the naturally engendered strain environment allows adaptive responses to artificial loading to be demonstrated at lower loads.

## Highlights

► Activity levels in mice housed in groups differ significantly; males tend to fight, females do not. ► In grouped males, the adaptive response to artificial loading at physiological strains is low suggesting pre-adaptation to fighting. ► When comparing adaptive responses to loading between groups of animals it is important to ensure activity levels are similar.

## Introduction

Bone architecture adapts to changes in mechanical strain engendered by its local functional loading environment [1]. This adaptation ensures that bones are sufficiently strong to withstand the mechanical loads they encounter without fracture or unsustainable levels of microdamage. To investigate the mechanisms underlying this adaptation, mouse models have been developed in which dynamic mechanical loads are applied *in vivo* to one limb, and adaptive changes to bone architecture measured and compared to the situation in contralateral non-loaded limbs [2–8]. Clearly, these artificially applied loads will only stimulate an adaptive response if the strains they engender differ significantly from those experienced during normal, day-to-day physical activity [8,9].

C57BL/6 mice are widely used for experimental studies since the majority of genetically modified mice are bred on this background [10]. In an initial pilot study the response to loading in male mice appeared inconsistent and markedly lower than that in females. Since this was unexpected [7,11] we investigated the behaviour of these mice. Differences in behaviour between group-housed males and females led us to perform the study we report here in which the response to unilateral tibial loading in animals housed individually was compared to that in animals housed in groups.

## Materials and Methods

### Animals

Sixteen-week-old male and female C57BL/6 mice were obtained from Charles River Inc. (Margate, UK) and, although prior to delivery they were housed in groups, fighting was reported to occur infrequently between males and not at all in females (personal communication). Within 24 h of arrival, five male and five female mice were sacrificed for *ex vivo* strain measurements (see later). Of the remaining animals, six males and six females were housed in individual cages and six of each sex were kept as a single group for five days before the study commenced. All mice were allowed free access to water and a maintenance diet containing 0.75% calcium (EURodent Diet 22%; PMI Nutrition International, LLC, Brentwood, MO, USA) in a 12-hour light/dark cycle, with room temperature at 21 ± 2 °C. All cages contained wood shavings, bedding and a cardboard tube for environmental enrichment. For one hour directly preceding each episode of *in vivo* loading, grouped mice were observed and any aggressive behaviour or fighting was recorded. The hour during which mice were observed was always at the same time of day in the morning, one hour after the start of the light period, by the same observer (LBM). All procedures complied with the UK Animals (Scientific Procedures) Act 1986 and were reviewed and approved by the University of Bristol ethics committee (Bristol, UK).

### *Ex vivo* Strain Measurements

To apply similar magnitudes of peak strain in male and female mice, we first established the strain:load relationship *ex vivo* in the sub-sample of five male and five female mice. In each mouse, a single element strain gage (EA-06-015DJ-120, Vishay Measurement Group, NC) was bonded longitudinally to the medial aspect of the tibia at 37% of its length from the proximal end. This is the site where we have previously observed the greatest osteogenic response to axial loading [12]. Strains were measured across a range of peak loads between 5 and 17 N, applied using the same electromagnetic loading machine used for *in vivo* loading (ElectroForce 3100; Bose Co., Eden Prairie, MN, USA). Linear regression analysis allowed calculation of the loads required to apply 2200 με at the start of the study.

### *In vivo* External Mechanical Loading

Right tibiae were subjected to external mechanical loading under isoflurane-induced anesthesia on alternate days for two weeks. Left limbs were used as internal controls as previously validated [12,13]. The protocol for non-invasively loading the mouse tibia has been reported previously [5,8,12]. In brief, the flexed knee and ankle joints are positioned in concave cups; the upper cup, containing the knee, is attached to an actuator arm and the lower cup to a dynamic load cell. The tibia is held in place by a 0.5 N continuous static pre-load. In this study, 40 cycles of dynamic load were superimposed with 10 s rest intervals between each cycle. The protocol for one cycle consisted of loading to the target peak load, hold for 0.05 s at the peak load, and unloading back to the 0.5 N pre-load. From the strain gage data (see “*ex vivo* strain measurements”), peak loads of 13.3 N for males and 13.0 N for females were required to engender 2200 με on the medial surface of the tibia. Strain rate at this site was normalized to a maximum of 30,000 μεs^− 1^ by applying the load at rates of 460 N/s in males and 450 N/s in females.

### High-resolution μCT Analysis

Following sacrifice, lower legs were stored in 70% ethanol and whole tibiae imaged using the SkyScan 1172 (SkyScan, Kontich, Belgium) with a voxel size of 4.8 μm (110 μm^3^). The scanning, reconstruction and method of analysis has been previously reported [8,14]. We evaluated the effect of housing and sex on both tibiae and changes [(right–left)/left] due to loading in bone volume fraction (BV/TV), trabecular thickness (Tb.Th), trabecular separation (Tb.Sp) and trabecular number (Tb.N) in the trabecular region (0.25–0.75 mm distal to the proximal physis) and cortical bone area (Ct.Ar), total cross-sectional area inside the periosteal envelope (Tt.Ar), medullary area (Ma.Ar), cortical area fraction (Ct.Ar/Tt.Ar), cortical thickness (Ct.Th) and polar moment of inertia (J), a parameter of structural bone strength, at the cortical site (37% from the proximal end), according to ASBMR guidelines [15].

### Serum Analyses

Three days after the final anesthesia/loading session, animals were euthanized by asphyxiation with carbon dioxide prior to cardiac puncture to minimize changes in corticosterone. Serum was separated by centrifugation and stored at − 80 °C until the time of analysis. Serum testosterone was measured using a competitive binding assay kit (R&D systems, MN) following manufacturers' instructions. Serum corticosterone was assayed using a competitive radioimmunoassay (Cort RIA, Izoto, Hungary) as previously described [16].

### Statistical analysis

The effect of housing, sex and their interaction on each bone parameter was assessed using a two-way ANOVA with interaction. When interactions were found to be significant, post-hoc t-tests were used for pair-wise comparisons to further examine the effect of housing within each sex. The effect of loading was assessed using paired samples t-tests. Differences in fighting and serum hormones were assessed using independent samples t-tests. Significance was set at p < 0.05. Analyses were performed using SPSS (version 18.0; SPSS Inc., Chicago, USA).

## Results

Tibial length and bodyweight in both male and female mice were not significantly different between grouped or individual mice at the end of the experiment (Table 1). Fighting was recorded 1.3 ± 0.5 times during the one-hour observation periods preceding mechanical loading in grouped male mice and never observed in females. This difference in the number of fights between groups was statistically significant (p < 0.05). Fighting in grouped males consisted of brief flurries of activity, usually involving two or three individuals at any one time. All males were seen to be involved in fights at least once during the observation period. No injuries were observed as a result of these episodes.

### Females

**Fig. 1:**
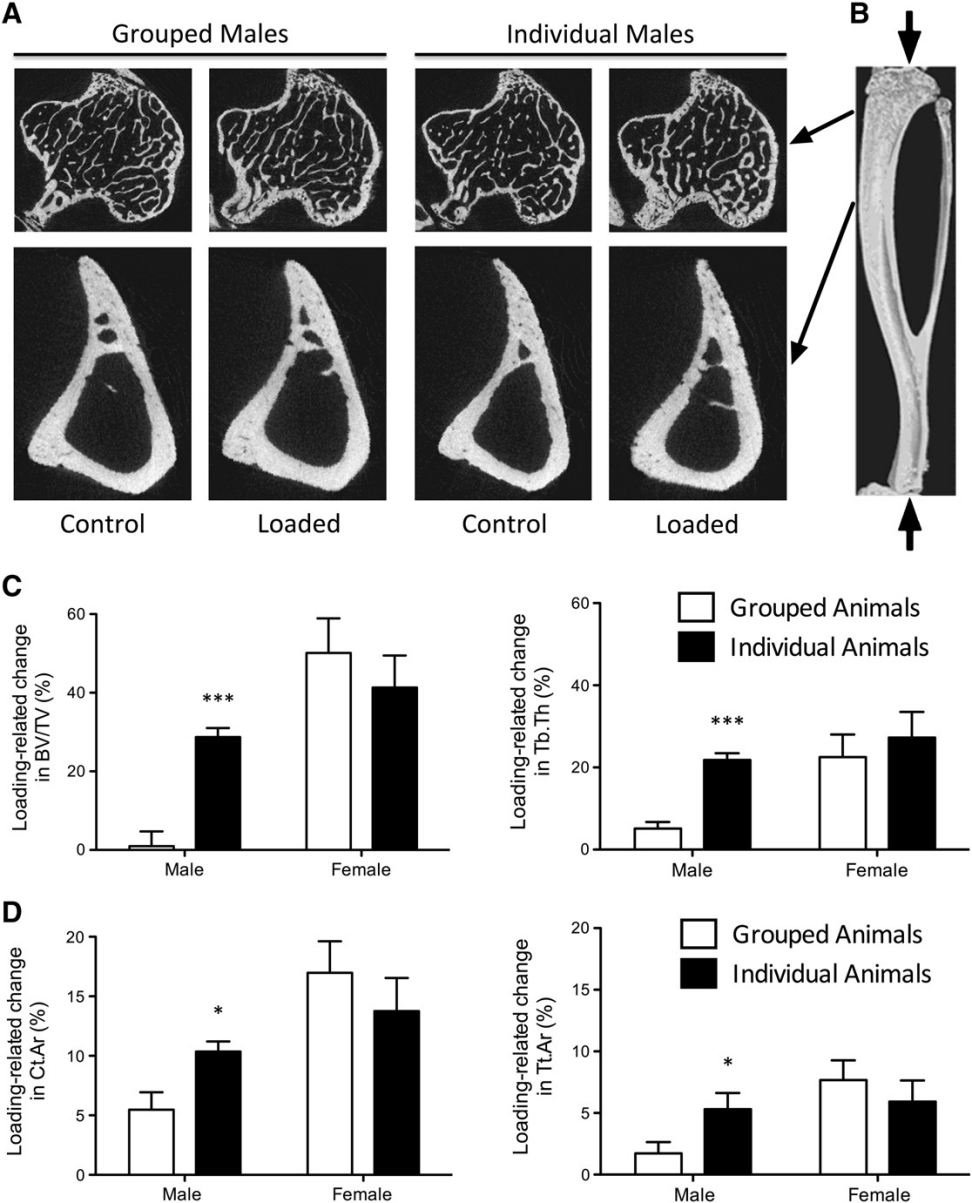
Group housing stimulates an adaptive osteogenic response, masking the effect of artificial loading, in the tibiae of male but not female mice. A: Representative μCT images of trabecular and cortical regions of the left control and right loaded tibiae in male mice. B: Representative 3D reconstruction of the mouse tibia; arrows indicate the direction of artificial loading. C & D: The effect of individual housing on the percentage change [(right–left)/left x 100], due to loading in trabecular (C) and cortical (D) bone compartments in male and female mice. Data represented as mean + SEM. BV/TV = bone volume fraction; Tb.Th = trabecular thickness; Ct.Ar = cortical bone area; Tt.Ar = total cross-sectional area inside the periosteal envelope. Asterisks indicate a significant difference due to housing within each sex: *p < 0.05, ***p < 0.001. The apparent response to loading was reduced in grouped, relative to individual, male mice.

There were no significant differences between left control tibiae from grouped or individual females for any parameter measured in trabecular or cortical bone (Table 1). In trabecular bone, loading significantly increased trabecular BV/TV, primarily due to an increase in Tb.Th. In cortical bone, Ct.Ar was significantly higher in right limbs after loading primarily due to an increase in Tt.Ar with no significant difference in Ma.Ar. There were no significant differences in the response to mechanical loading between grouped and individual female mice (Fig. 1). Serum corticosterone concentration was not different between grouped and individual females (Table 1).

### Males

In contrast to females, the left non-loaded tibiae of grouped male mice had significantly higher trabecular BV/TV (28.6% higher than individual male mice, p < 0.001, Table 1) due primarily to greater Tb.Th (19.0%, p < 0.01) and a smaller, but still significant difference in Tb.N (7.9%, p < 0.05). The left non-loaded tibiae of grouped males also had higher Ct.Ar (11.5%, p < 0.01) and Tt.Ar (12.5%, p < 0.05, Table 1). No difference in serum testosterone concentration was detected between grouped and individual males. However, somewhat surprisingly, grouped males had a significantly lower serum corticosterone concentration (− 59.4%, p < 0.05).

When loaded and non-loaded tibiae were compared in individual male mice, there was a highly significant difference in trabecular BV/TV (28.7%, p < 0.001) and Tb.Th (21.8%, p < 0.001). This difference was much less in grouped males (0.8%, p = 0.85 and 4.9%, p < 0.05 respectively, Fig. 1). In cortical bone, loading was associated with a significantly increased Ct.Ar in individual males (8.7%, p < 0.01), again associated with increased Tt.Ar (5.5%, p < 0.01). However, grouped males showed a smaller difference in Ct.Ar (5.4%, p < 0.05) and no difference in Tt.Ar (1.8%, p = 0.13) between loaded and non-loaded bones.

## Discussion

Data from our pilot experiment suggested that male C57BL/6 mice showed a lower osteogenic response to artificial loading than females, contradicting the results from previous studies demonstrating no such sex-related difference [7,11]. Closer examination of the conditions in which the separate sexes of mice were kept modified that initial interpretation, since the lower response to artificial loading was confined to grouped male mice. Examining the behavior of grouped males revealed no outstanding differences except that, unlike females, the males indulged in frequent fights which, while causing no apparent injuries, involved short periods of vigorous activity. These fights occurred primarily following delivery to our institution, possibly associated with the establishment of a new dominance hierarchy in a new environment.

Although measurements of strain during fighting were not attempted for welfare reasons, peak strains and strain rates up to 5000 με and 100,000 με s^− 1^ respectively have been previously recorded during vigorous activities in animals [17,18]. Therefore it is probable that the peak strains, and strain rates, engendered during fighting exceed those engendered during artificial loading. If this were so it would be expected to stimulate an adaptive increase in bone mass in both tibiae. It is well documented that only a few cycles of loading are sufficient to induce such an osteogenic response [19,20]. This number could easily be achieved during these brief and frantic periods of fighting. Neither individual males nor grouped or individual females indulged in fighting and thus were not exposed to this level of osteogenic stimulus. This is consistent with artificial loading producing a strain-related stimulus exceeding that provided naturally.

At the end of our experiment, higher measures of bone mass were observed in left, non-loaded, control limbs of grouped compared to individual males. This concurs with the findings of Nagy et al. [21], that growing mice housed in groups had a significantly higher bone mineral density and bone mineral content compared to those individually housed. One limitation of our study is the absence of quantitative histomorphometric analysis or *in vivo* μCT. However, qualitative analysis of fluorochrome labelled cortical bone sections indicated that bone formation was increased during the study period in the control limbs of group compared to individually housed males (data not shown).

Although androgen receptor signalling can affect bone's response to mechanical loading [22] and male mice who win fighting contests have previously been shown to have higher levels of testosterone [23], we found no significant difference in testosterone serum concentrations between grouped and individual males. It is possible that testosterone only increases transiently during episodes of fighting so, by sampling mice after sacrifice, we may not have detected these fluctuations. In addition to the effect of housing on testosterone, we examined whether housing affected serum corticosterone. Interestingly, we found that grouped males had significantly lower corticosterone than those housed individually. This is consistent with a previous study in which fecal corticosterone was reduced in grouped males with environmental enrichment, but not in individual males [24]. Nevertheless it is unlikely that a reduction in serum corticosterone in grouped males could account for bone's reduced response to mechanical loading, since increased levels of endogenous glucocorticoids are associated with reduced bone mass [25]. In contrast, there was no effect of housing conditions on serum corticosterone in female mice. In agreement with previous studies, corticosterone levels in female mice were higher than those in males [26].

In conclusion, the data presented in this study suggest that high levels of habitual background activity, associated in this case with fighting in male mice, may stimulate a sufficient increase in bone mass to negate any additional osteogenic effect of short periods of artificial loading at peak strain levels that safely avoid damage. This indicates the importance in studies of this type of ensuring that any stimulus provided by artificial loading is normalized for strains achieved rather than loads applied and that background physical activity levels of animals involved are similar between groups.

## Figures and Tables

**Table 1:** Bodyweight, tibial length, trabecular and cortical bone parameters measured using high-resolution μCT and serum analyses.

Sex	Male		Female	
Housing	Group	Individual	Group	Individual
Bodyweight (g)	30.1 ± 0.7	28.9 ± 0.7	22.0 ± 0.7	21.8 ± 0.4
Fighting (no./h)	1.3 ± 0.5	N/A	0 ± 0	N/A
Tibial length (mm)	18.3 ± 0.1	18.1 ± 0.2	17.7 ± 0.1	17.7 ± 0.1
Trabecular bone				
BV/TV (%)				
Left control	14.6 ± 0.5	**11.4 ± 0.3^c^**	6.8 ± 0.2	6.9 ± 0.9
Right loaded	14.7 ± 0.6	**14.6 ± 0.5^f^**	**10.2 ± 0.5^e^**	**9.8 ± 1.1^e^**
Tb.Th (mm)				
Left control	0.045 ± 0.002	**0.038 ± 0.001^b^**	0.047 ± 0.002	0.044 ± 0.003
Right loaded	**0.047 ± 0.001^d^**	**0.046 ± 0.002^f^**	**0.058 ± 0.002^e^**	**0.056 ± 0.001^d^**
Tb.Sp (mm)				
Left control	0.172 ± 0.001	0.170 ± 0.002	0.256 ± 0.009	0.241 ± 0.017
Right loaded	0.169 ± 0.002	0.166 ± 0.002	**0.242 ± 0.008^d^**	0.240 ± 0.012
Tb.N (mm^− 1^)				
Left control	3.25 ± 0.06	**3.01 ± 0.06^a^**	1.44 ± 0.06	1.60 ± 0.29
Right loaded	3.11 ± 0.10	**3.18 ± 0.02^d^**	**1.77 ± 0.12^d^**	1.74 ± 0.22
Cortical bone				
Ct.Ar (mm^2^)				
Left control	0.85 ± 0.01	**0.76 ± 0.02^b^**	0.68 ± 0.02	0.66 ± 0.02
Right loaded	**0.89 ± 0.01^d^**	**0.83 ± 0.02^f^**	**0.79 ± 0.02^f^**	**0.74 ± 0.002^d^**
Tt.Ar (mm^2^)				
Left control	1.52 ± 0.03	**1.35 ± 0.04^a^**	1.17 ± 0.04	1.15 ± 0.03
Right loaded	1.55 ± 0.04	**1.43 ± 0.03^e^**	**1.26 ± 0.03^e^**	**1.22 ± 0.02^d^**
Ma.Ar (mm^2^)				
Left control	0.67 ± 0.03	0.59 ± 0.03	0.49 ± 0.02	0.50 ± 0.01
Right loaded	0.65 ± 0.03	0.60 ± 0.02	0.47 ± 0.01	0.48 ± 0.02
Ct.Ar/Tt.Ar (%)				
Left control	55.8 ± 0.8	56.3 ± 0.7	58.1 ± 0.4	57.0 ± 0.9
Right loaded	57.8 ± 1.0	**58.0 ± 1.0^e^**	**62.6 ± 0.6^e^**	**60.9 ± 0.8^d^**
Ct.Th (mm)				
Left control	0.147 ± 0.002	0.142 ± 0.002	0.143 ± 0.003	0.140 ± 0.004
Right loaded	**0.160 ± 0.003^e^**	**0.153 ± 0.002^e^**	**0.160 ± 0.004^f^**	**0.159 ± 0.003^d^**
J (mm^4^)				
Left control	0.439 ± 0.015	**0.365 ± 0.015^b^**	0.267 ± 0.017	0.254 ± 0.011
Right loaded	0.452 ± 0.016	**0.398 ± 0.018^e^**	**0.321 ± 0.015^e^**	**0.296 ± 0.007^d^**
Serum				
Testosterone (ng/ml)	4.29 ± 6.28	3.81 ± 3.31	–	–
Corticosterone (ng/ml)	89.1 ± 15.6	**187.2 ± 37.4^a^**	298.5 ± 74.0	265.9 ± 42.5

Data represented as mean ± SEM (male and grouped female n = 6; individual female n = 4). BV/TV = bone volume fraction; Tb.Th = trabecular thickness; Tb.Sp = trabecular separation; Tb.N = trabecular number; Ct.Ar = cortical bone area; Tt.Ar = total cross-sectional area inside the periosteal envelope; Ma.Ar = medullary area; Ct.Ar/Tt.Ar = cortical area fraction; Ct.Th = cortical thickness; J = polar moment of inertia. ^a^p < 0.05, ^b^p < 0.01, ^c^p < 0.001 individual compared to group housed mice of the same sex; ^d^p < 0.05, ^e^p < 0.01, ^f^p < 0.001 comparing left control with right loaded limbs.
